# Photopolymerization of Styrene–Naphthalenediimide Monomer: Formation of Pattern and Electrochromism

**DOI:** 10.3390/ijms26104807

**Published:** 2025-05-17

**Authors:** Marcin Nowacki, Marcin Hoffmann, Monika Wałęsa-Chorab

**Affiliations:** Faculty of Chemistry, Adam Mickiewicz University in Poznań, Uniwersytetu Poznańskiego 8, 61-614 Poznań, Poland; marcin.nowacki@amu.edu.pl (M.N.); mmh@amu.edu.pl (M.H.)

**Keywords:** naphthalenediimide, electrochromism, photopolymerization, electrochromic device

## Abstract

The electrochromic naphthalenediimide (NDI) based monomer containing styrene pedant groups, which are capable of polymerization, was prepared, and the formation of its polymer via a photopolymerization reaction was described. Both the monomer and polymer exhibited a color change in the visible range from transparent or slightly yellow, respectively, followed by brown-red to green. This was the result of a two-step reduction reaction of NDI core to radical anion and dianion, respectively. The device constructed using the polymer as an active material was found to exhibit good electrochromic stability over 500 redox cycles. The switching times were calculated to be 18 s and 6 s for the coloration and bleaching steps, respectively. The presented results showed the usability of the photopolymerization of styrene-based monomers in the generation of the stable electrochromic layers of polymers.

## 1. Introduction

Electrochromic compounds are one of the groups of responsive materials, which are able to change their coloration in response to an external electric impulse [1]. Under such stimulus, the oxidation or reduction of the material occurs, resulting in a visible color change. Depending on the electrochromic material, a change from one color to another [2,3,4] or a colorless-to-color [5,6,7] state can be observed, and the switching between two different color states can be performed reversibly. Such intelligent color-changing systems can potentially be applied in a wide range of different research areas, such as electrochromic artificial skin [8,9], smart windows [10,11], or electrochromic displays [12,13].

Electrochromic displays have many advantages [14,15]. They operate in the subtractive (or light-absorbing) color mode, and due to this, such displays are eye-friendly and have ideal outdoor readability during the day. This is because there is no radiation of blue light, which may have an adverse effect on human eyes. Most existing electrochromic compounds are known to have good compatibility with numerous different substrates, such as plastic [16], metal [17], fibers, and even different textiles [18,19], which enables the construction of flexible displays. In comparison with other displays, electrochromic displays exhibit a low consumption of electrical energy due to the optical memory effect. This means that such materials can remain in the appropriate optical state for some time without a continuous supply of an electrical current [20,21].

One of the groups of electrochromic displays are electrochromic segmented displays, which represent a relatively simple display modem which enables the display of letters, numbers, or graphics. A major step in the construction of electrochromic segmented displays is the formation of an appropriate pattern. One strategy to achieve this is active material patterning, which involves various techniques such as photolithography [22,23], printing [24,25], or other template-assisted methods [26,27]. Photolithography, also called UV lithography or optical lithography, is a method in which the layer of the material is covered with a photomask and is irradiated with the light of an appropriate wavelength [28]. This enables the transfer of a graphic pattern from a photomask to a substrate through a specific chemical process that depends on exposure to light of the appropriate wavelength. Prominent examples of such photo-driven chemical reactions are radical-based thiol–ene click reaction [29], cationic ring-opening polymerization of oxetanes [30], or catalyst-free spontaneous polymerization of disulfonic acids and dihaloalkynes [31]. The advantage of using a photomask is the capability to pattern arbitrary shapes, e.g., shapes that are frames with fill inside, which is difficult to achieve with the spray-coating technique using a template.

**Figure 1:**
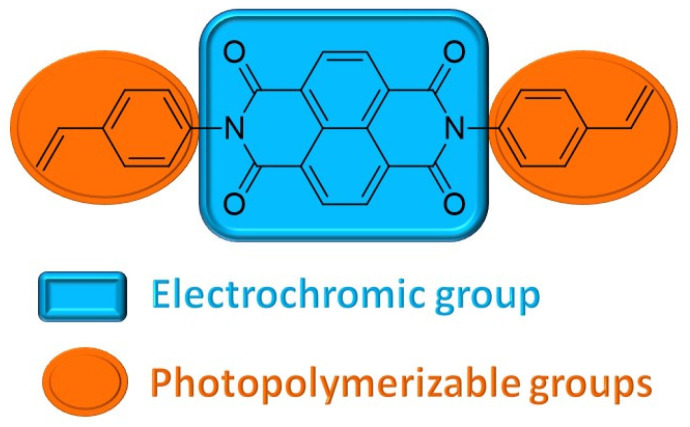
The structure of the NDI–styrene monomer.

Previously, we have presented that styrene derivatives are useful monomers in the photopolymerization reaction [32]. In general, the polymerization reactions of vinyl monomers, including styrene derivatives, are radical-initiated reactions. The radicals can be formed from styrene [27,33,34] or some radical initiator [35,36,37] in a thermal polymerization process. The polymerization of styrene derivatives can also be performed electrochemically via reductive electropolymerization [38,39,40]. The photopolymerization reaction of styrene derivatives requires the addition of a catalytic amount of 2,2-dimethoxy-2-phenylacetophenone (DMPA) that upon exposure to UV light forms radicals, which in turn causes the polymerization reaction of styrene groups. The obtained previously viologen-based polymers exhibited electrochromism, but their electrochromic stability was not explored. The electrochromic material should exhibit stability during multiple oxidation/reduction cycles, and poor stability may result from the degradation of the electroactive group or delamination of the polymer from the electrode surface. In light of this, we designed a monomer containing naphthalenediimide (NDI) central moiety, which is known to exhibit a distinct color change during redox processes and styrene pedant groups able to undergo photopolymerization (Figure 1). The NDI fragment is also characterized by good electrochromic stability, which allowed us to investigate the suitability of the photopolymerization method for electrochromic applications.

During the electrochemical process, NDI-based materials undergo two one-electron reduction processes, which are ascribed to the reversible [NDI]↔[NDI]^−^ and [NDI]^−^↔[NDI]^2−^ redox reactions [41,42]. NDI-based materials are very interesting for electrochromic applications [43,44,45,46,47]. These reductions are associated with distinct color changes due to the generation of radical anion and dianion forms of NDI. AlKaabi and co-workers reported electrochromic NDI-based metal-organic framework (MOF) films exhibiting well-behaved quasi-reversible redox processes responsible for a transparent to dark electrochromic switching [48]. Hsiao et al. [49] designed and prepared the NDI–triphenylamine monomer, which, under applied potential, polymerized onto the electrode substrate, forming a multielectrochromic polymer. The straightforward synthesis of NDI derivatives via condensation of the desired amine with naphthalene dianhydride allows for precise tuning of the electronic and structural properties.

The obtained monomer was photopolymerized on the indium tin oxide coated glass slides (ITO), and the electrochromic performance of the photopolymerized layer was investigated to show the usefulness of the photopolymerization protocol in the construction of electrochromic devices.

## 2. Results and Discussion

### 2.1. Synthesis of the Monomer

Naphthalenediimide derivatives can be obtained in a straightforward reaction between 1,4,5,8-naphthalenetetracarboxylic dianhydride and the desired amine. The NDI monomer was obtained in the condensation reaction, as outlined in Figure S1. The targeted styrene–NDI monomer was prepared in the microwave-assisted condensation of 1,4,5,8-naphthalenetetracarboxylic dianhydride (NDA) with 4-vinylaniline [50]. The reaction was carried out in a microwave reactor using a specially designed reaction vessel, which was tightly closed and pressure-resistant. Microwave-assisted synthesis has many advantages over conventional organic synthesis. It allows for precise heating control, and due to this, compounds can be prepared in a more selective way. Many organic chemical reactions are accelerated by microwave irradiation, and the selection of appropriate microwave synthesis conditions leads to increased reaction selectivity [51]. The condensation was conducted at 160 °C for 90 min in dimethylformamide (DMF), and the slightly dark-colored product was precipitated using 1 M of HCl, filtrated, rinsed a few times with distilled water, and dried in air. Furthermore, the material was fully characterized using spectroscopic techniques (Figures S2–S4), and the obtained analytical data were consistent with the chemical structure of the monomer and confirmed its purity. In ^1^H NMR spectra, two doublets at 5.38 ppm and 5.96 ppm and a doublet of doublets at 6.85 ppm are characteristic of a vinylic double bond [35], and their presence in ^1^H NMR spectra (Figure S2) confirms that the styrene unsaturated bonds tolerated the synthesis conditions. The singlet at 8.71 ppm is characteristic of the four protons of naphthalene core and proves that the final product is symmetrically substituted, while two doublets at 7.66 ppm and 7.44 ppm have been assigned to protons from para-substituted benzene rings.

### 2.2. Photopolymerization

In the next step, the NDI–styrene monomer was subjected to a photopolymerization reaction. To achieve this, the solution of a monomer in a dichloromethane/methanol solvent mixture of 5:1 (*v*/*v*) was prepared and 2,2-dimethoxy-2-phenylacetophenone (DMPA) (10% mol) was added to the solution. It was observed that the monomer at this concentration (4 mg of the monomer in 2 mL of dichloromethane) was only partially soluble in pure dichloromethane. The addition of methanol (0.4 mL) to the suspension of the compound in dichloromethane resulted in solubilization of the monomer. As is known, the NDI derivatives can form a π–π stacked structure in the aggregated state [52,53] and can also form C–H···O hydrogen bonds between the aromatic protons of the NDI unit and the carbonyl oxygen atoms [54]. The addition of methanol probably caused a disruption of these interactions, leading to better solubility of the compound. DMPA was chosen as a photoinitiator of the reaction because it is known to undergo photolysis when irradiated with UV light to form radicals [55] that are capable of initiating the polymerization reaction of styrene. Afterwards, the mixture of monomer and initiator was deposited on the indium–tin oxide coated glass slides (ITO) using a spray-coating method, and the substrates were placed in the multi-wavelength PhotoCube photoreactor and irradiated with UV-light (365 nm) for 10 min. Four LED panels were used, and the light intensity was set to be 100% (radiant flux 44.8 W). This allowed us to obtain homogenous irradiation of the sample. It was found that the presence of oxygen and moisture does not interfere with the photopolymerization reaction; therefore, it is possible to carry out the reaction in a normal atmosphere without the need to use inert gases. The polymerized layers were rinsed with dichloromethane a couple of times to wash out the unreacted monomer and low-molecular-weight products and then air-dried. The result was a yellow-colored film, which was physically absorbed on the substrate surface. The solubility of the polymer was investigated in different polar and nonpolar solvents as well as dichloromethane/methanol solvent mixtures of 5:1 and 1:1 (*v*/*v*) (Table S1). The substrate coated with the polymer was placed in 5 mL of solvent and stirred for 10 min. UV-Vis absorption spectra of the solution were then recorded to check whether the polymer had been dissolved. The obtained polymer has been found to be insoluble in commonly used organic solvents, which is an advantage when used as an active material in electrochromic devices.

**Figure 2:**
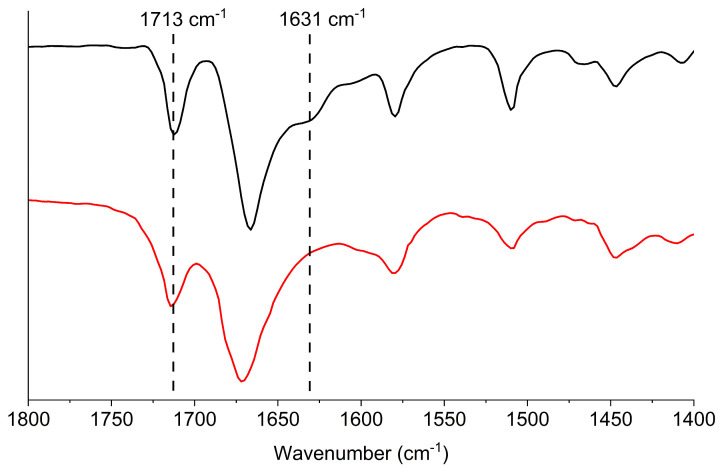
ATR-FT-IR spectra of NDI–styrene monomer (black) and polymer (red).

The photopolymerization reaction was monitored by infrared spectroscopy (Figure 2). It can be seen that the peak at 1631 cm^−1^ corresponding to the stretching vibrations of a carbon–carbon double bond vinyl group from styrene moiety disappeared, which confirms the successful photopolymerization reaction [27,56]. On the other hand, the vibration at 1713 cm^−1^ assigned to the six-membered imide group remains unchanged, indicating that the NDI core is stable during the photopolymerization reaction [56].

The thermal stability of the monomer and polymer was investigated using thermal gravimetry analysis (TGA). The TGA results (Figure S5) demonstrate that both materials exhibit sufficient thermal stability comparable to other NDI derivatives [57,58]. The decomposition temperature of the monomer was found to be in the range of 230–305 °C, while the polymer shows an onset decomposition at 295 °C, demonstrating that the polymer has better thermal stability compared to the monomer. The small weight loss up to 100 °C was probably due to the loss of adsorbed water because the measurement was performed without a prior thermal treatment.

**Figure 3:**
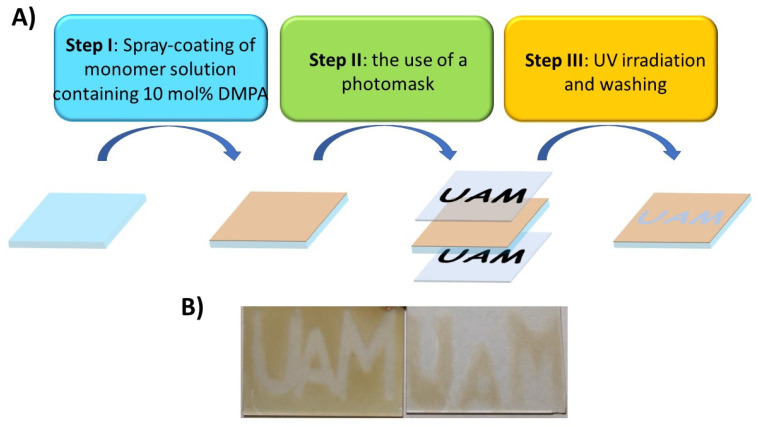
(**A**) Scheme showing the formation of photopattern; (**B**) photograph showing the photopatterns photolithographically immobilized on a glass substrate.

Furthermore, the formation of patterns was also investigated. This was performed in three steps, as outlined in Figure 3A. First, the layer of the monomer containing 10 mol % of DMPA was deposited on one side of the glass substrate using spray-coating. Next, the substrate coated with the thin layer of spray-coated NDI–styrene compound was covered with the photopattern on the glass slide with the marked inscription. Because the UV-irradiation of the substrate in the used photoreactor occurs both on the top and bottom of the sample, a second, identical photomask was placed under the sample. This way, the substrate with the layer of monomer was covered by the photomasks from the top and bottom. Then, it was irradiated with UV light; the obtained polymer layer was rinsed with dichloromethane and dried. As can be seen in Figure 3B, polymerization did not occur in places which were covered by the photomask, and this confirms that styrene-based monomers can be used in the formation of patterns for electrochromic segmented displays.

### 2.3. DFT Calculations

Quantum chemical calculations of the electronic and geometrical structure of the NDO–styrene monomer were performed using the density functional theory (DFT) with the widely adopted B3LYP hybrid functional [59,60]. The basis set employed was 6-31+G(d,p), which includes both diffuse and polarization functions [61,62]. This level of theory was selected as a compromise between computational efficiency and the desired accuracy. As shown in our previous studies, the inclusion of diffuse functions was essential for an appropriate description of the anionic species [63,64,65,66]. All of the calculations were carried out using the Gaussian 16 software package [67]. The optimized molecular geometry is shown in Figure S6, along with the corresponding Cartesian coordinates (Table S2). The naphthalene core adopts a planar conformation, while the styrene substituents are oriented perpendicularly, resulting in a stable structure consistent with those reported for other NDI-based systems [68,69].

To gain a deeper insight into the optical absorption characteristics of the NDI–styrene monomer, we conducted time-dependent density functional theory (TDDFT) calculations [70,71]. Utilizing the B3LYP functional in conjunction with the 6-31+G(d,p) basis set, these computations enabled the prediction of vertical excitation energies and oscillator strengths, thereby facilitating the simulation of the UV-Vis absorption spectrum. The TDDFT approach is particularly effective for modeling excited-state properties and has been widely employed to study the optical behavior of organic semiconductors [72] and donor–acceptor systems [73,74].

**Figure 4:**
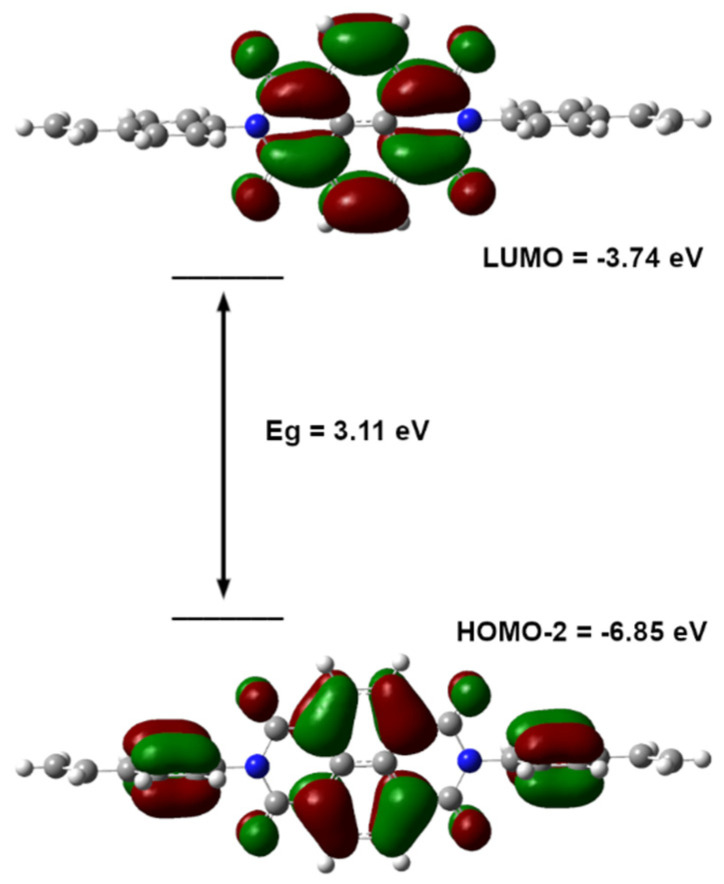
Molecular orbitals involved in the photon absorption by NDI–styrene monomer.

The results indicate that the quantum chemically derived spectrum (Figure S6) corresponds very well with the experimental measurements and exhibits absorption peaks at 3.11 eV (398.5 nm) and 3.32 eV (373.7 nm). Indeed, the role of HOMO–LUMO localization in electrochromism is speculative, but the TD-DFT-calculated electronic transitions indicate that the excitation from the HOMO-2 to LUMO orbital corresponds to the observed light absorption (see Figure S6). The accepting LUMO orbital is localized in the naphtalenediimidine core, while the donating HOMO-2 orbital is also localized in the styrene rings (Figure 4).

For the comparison, the experimentally obtained energy gap for the ultraviolet–visible light absorption spectra was calculated in accordance with the following equation:

$$E_{g(opt.)}\left(eV\right)=\frac{1240}{\lambda (nm)}$$

where E_g(opt.)_ is the optical band gap in eV, and λ indicates the absorption limit wavelength in nm. The value of the absorption edge was obtained from the offset wavelength originating from the low-energy absorption band (Figures S7 and S8) [75]. For both NDI–styrene monomer and NDI-based polymer, an identical optical band gap value of 3.11 eV was estimated. This was consistent with the values of the optical band gap energies obtained for similar NDI derivatives [76,77] and indicates that the polymerization of the styrene groups does not change the optical band gap. The LUMO levels of the monomer and polymer were also calculated from cyclic voltammetry (vide infra) according to the following equation:

E_LUMO_ = −(E_pc onset_ + 4.80) [eV]

The onset potentials were found to have the same value (−0.85 V vs. ferrocene/ferrocenium couple) for the monomer and polymer, and the calculated LUMO levels were −3.95 eV for both compounds, which is consistent with the theoretical calculations.

### 2.4. Electrochemistry and UV-Vis Spectroelectrochemistry

**Figure 5:**
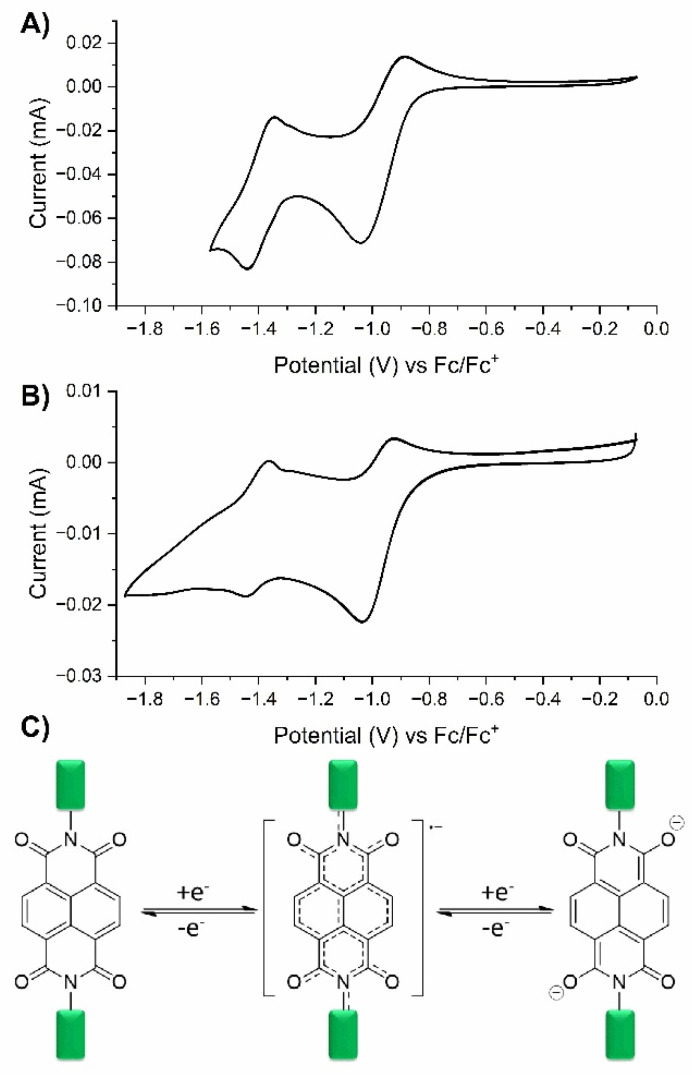
(**A**) Cyclic voltammetry curve for the NDI–styrene monomer; (**B**) cyclic voltammetry curve for the NDI-based polymer measure at scan rate 100 mV/s; (**C**) mechanism of reduction of NDI-based compounds to radical anion and dianion.

The electrochemistry of the monomer was investigated in the solution in the three-electrode configuration using the platinum electrode (ϕ = 2 mm) as a working electrode, whereas the non-aqueous Ag/Ag^+^ electrode and platinum wire were used as reference and counter electrodes, respectively. For the polymer measurements, the ITO electrode coated with the polymer was used as a working electrode. The measurements were performed in solution of TBAClO_4_ (0.1 M) in acetonitrile as a supporting electrolyte. Prior to the measurements, the reference electrode was calibrated using an external standard (ferrocene), and the cyclic voltammograms were calibrated against the ferrocene/ferrocenium (Fc/Fc^+^) redox couple. Figure 5A shows the cyclic voltammogram of the NDI-based monomer, and it can be seen that the monomer undergoes two consecutive quasi-reversible redox processes at potentials of −1040 mV and −1440 mV, which are associated with the oxidation potentials at −880 mV and −1350 mV, respectively, in the reverse scan. Such behavior is typical for NDI derivatives and is associated with the generation of a radical anion in the first step and the transition of an anion radical to dianion in the second step (Figure 5C) [78]. Similar electrochemical behavior was observed for the polymer. The reduction potentials were found to be at −1035 mV and −1446 mV for NDI→NDI^−^ and NDI^−^→NDI^2−^, respectively (Figure 5B).

To investigate the character of the electrochemical processes of the polymer, the cyclic voltammograms of the polymer were recorded in the potential range of the first reduction process (Figure S9). As shown in Figure S10, the cathodic current of the first reduction peak increased proportionally with the scan rate with the linear regression coefficient R^2^ = 0.997. This confirms that the polymer adheres to the electrode surface and that the electrochemical events occur at the electrode surface.

**Figure 6:**
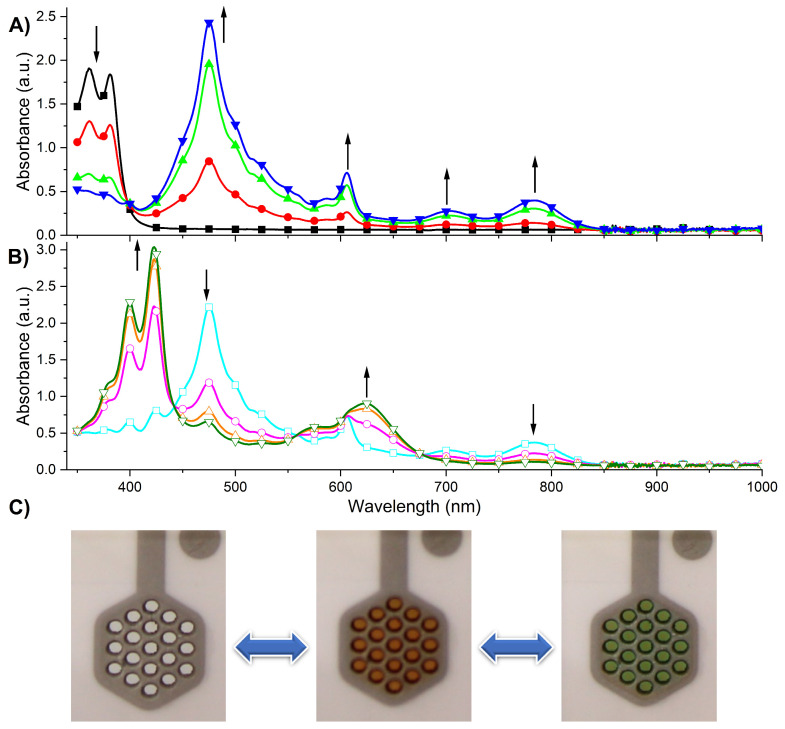
UV-Vis spectroelectrochemistry of NDI–styrene monomer measured with applied potentials of (**A**) 0V (◼), −0.9 V (●), −1.0 V (▲), and −1.1 V (▼); (**B**) −1.2 V (□), −1.3 V (○), −1.4 V (Δ), and −1.5 V (∇); (**C**) photographs of the monomer in its neutral form (**left**), in the form of radical anion (**middle**), and in the form of dianion (**right**).

Considering the reversible redox reaction of the NDI-based monomer, we attempt to investigate its electrochromic properties. The UV-Vis spectroelectrochemistry of the NDI-based monomer has been investigated using a platinum honeycomb electrode. The series of UV-Vis spectra have been measured by applying the reducing potentials in the range from 0 V to −1.5 V. As seen in Figure 6A, the absorption spectrum of the NDI-based monomer in its neutral state is characterized by two absorption maxima at 360 and 381 nm, and the spectrum shows the vibronic structure, which is typical for the π−π* transition of the NDI core [79]. The intensity of these bands gradually decreases as the applying potential becomes more negative up to −1.1 V. At the same time, the new absorption bands at 475 nm, 606 nm, 703 nm, and 785 nm appeared, which are characteristic of the NDI radical anion [79,80]. The color change from colorless to brown-red (Figure 6C) has been noticed. At more negative potentials, the intensity of the bands observed for the radical anion decreased, and new absorption bands centered at 400 nm, 423 nm, and 625 nm appeared (Figure 6B). The obtained spectra are characteristic of the NDI dianion. The color change was from brown-red to green. The distinct isosbestic points at 398 nm for NDI→NDI^−^ transition and at 442 nm and 558 nm for NDI^−^→NDI^2−^ electrochemical reaction have been observed. Similar optical changes have been observed for the NDI-based polymer (Figure S11).

### 2.5. Electrochromic Device

To evaluate the usefulness of the behavior of the photopolymerized thin layer of the NDI-based monomer in an operating electrochromic device, prototype absorptive/transmissive window-type electrochromic devices have been fabricated. This allowed us to fully test the capacity of the polymer for color change over multiple redox cycles, the speed of the color change, as well as the coloration efficiency. For this, the monomer was deposited on the ITO electrode, and it was photopolymerized according to the method described above. The modified ITO electrode has been used for the construction of the color-changing device of the active area of 3 cm^2^ (2 cm × 1.5 cm).

**Figure 7:**
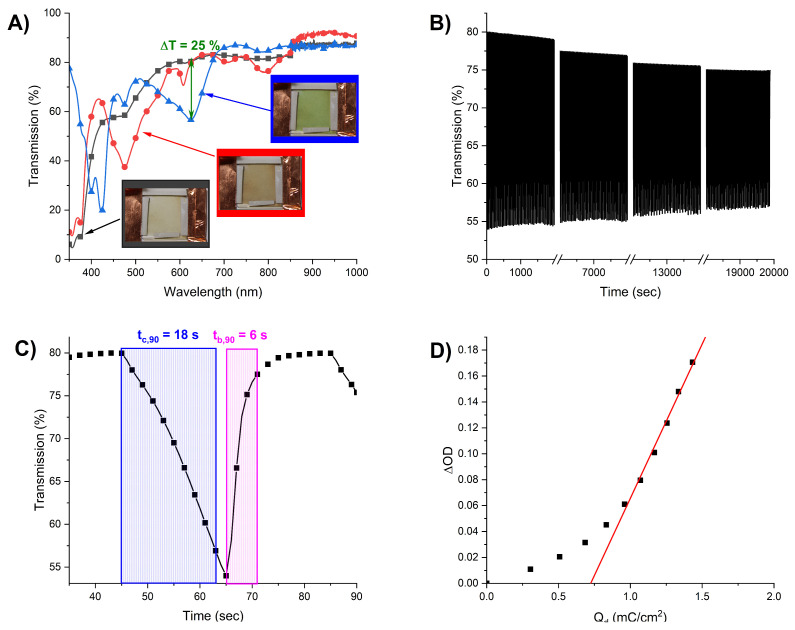
(**A**) The transmission spectra of the device fabricated using NDI-based polymer after applying the potentials of 0 V (■), −1.5 V (●), and −2.0 V (▲); green arrow indicates the transmission difference between neutral and fully reduced states. Insert: the photographs of the device after applying the potentials of 0 V (black frame), −1.5 V (red frame), and −2.0 V (blue frame); (**B**) change in percent transmission of the device at 625 nm with applied potentials of −2.0 V and +0.5 V switched at 20 s intervals; (**C**) response times of the device; (**D**) the plot of optical density vs. charge density per unit electrode area necessary to induce appropriate transmission change.

The device assembled using the NDI-based polymer as an active material undergoes a two-step color change depending on the applied potential. The colors observed for the electrochromic device were similar to the colors of the monomer in the appropriate redox state in the solution. As can be seen in Figure 7A, the transmission spectra of the electrochromic device in both the colored states and the bleached state were obtained in the entire visible area from 380 to 1000 nm.

High transmission was observed when the polymer was in its neutral state. This was consistent with the observed yellow color. When the reduction potential of −1.5 V was applied to the device, the color change in the polymer to orange-brown was noticed. This was connected with the transmission decrease in the blue-green range of the spectrum (~420–550 nm). Applying the more negative potential (−2.0 V) resulted in the color changing from orange-brown to green. During this process, the transmission of the device decreased in two regions: in the violet/indigo region between 380 and 440 nm and in the yellow/orange region between 550 and 660 nm. This is consistent with the subtractive color mixing, where the green color can be obtained by mixing at least two chromophores that absorb light in the yellow and blue regions of the visible spectrum [81]. The color changes were found to be reversible. Applying the slightly oxidizing potential (+0.5 V) resulted in the restoration of the original neutral state.

The stability of the device for a certain period of time was examined by switching the potential between −2.0 V and +0.5 V in 20 s intervals. This corresponds to a green ↔ yellow color change. The transmission of the device was monitored at 625 nm. The performance of the device is shown in Figure 7B. It can be seen that the device could operate over 500 oxidation/reduction cycles. At the beginning of the operation, the transmission percent difference (ΔT%) between the original and reduced states was ~26%, and after 500 redox cycles, ΔT% dropped down to ~20%. This is probably the result of the polymer degradation and delamination from the electrode.

To further examine the device’s performance, the response time and coloration efficiency were evaluated. The coloration (t_c,90_) and bleaching (t_b,90_) times have been defined as the time required to reach 90% coloration between two redox states: colored and bleached ones (Figure 7C). The measured response times were 18 s for the coloring step and 6 s for bleaching. Figure 7D shows the plot of optical density (ΔOD) versus charge density (Q_d_) necessary to induce an appropriate transmission change. The optical density of the polymer has been calculated as the logarithm of the ratio of transmission value in bleached (T_b_) and colored (T_c_) states, respectively:

$$∆OD=log(\frac{T_{b}}{T_{c}})$$

The value of the coloration efficiency of the NDI-based polymer was 238 cm^2^/C, and it has been extracted as the slope of the line fitted to the linear region of the curve [82,83].

To test the color memory of the device, it was reduced to the green color, and the potential was turned off. The change in the transmission of the device over time is shown in Figure S12. It can be seen that the transmittance of the device decreased by around 50% after 40 s and by around 90% after 660 s. This could be improved by adding an additional ion-storage layer [84].

The electrochromic performance of the electrochromic device constructed using NDI-based polymer was found to be comparable to other NDI-based electrochromic materials. This is evident from the compiled coloration and bleaching times as well as transmission contrast and coloration efficiencies summarized in Table 1. This gives it significant potential in electrochromic applications.

## 3. Materials and Methods

Spectroscopic measurements consisted in performing NMR spectra using the Bruker Avance 600 MHz apparatus (Karlsruhe, Germany). The tests were performed in deuterated solvents from Deutero GmbH (Kastellaun, Germany), and the spectra were calibrated against the solvent signal (DMSO = 2.50 ppm). EI-MS spectra was measured on a Spectrometer AMD Intectra Mass AMD 402 (Harpstedt, Germany). Electrochemical measurements were done using a multichannel Bio-Logic VSP potentiostat (Seyssinet-Pariset, France) in 0.1 M solution of tetrabutylammonium perchlorate (TBAClO_4_) in acetonitrile as a supporting electrolyte. The acetonitrile was dried by passing over the neutral alumina and stored over 3 Å molecular sieves prior the preparation of the electrolyte [87]. The solution for electrochemical and spectroelectrochemical measurements was purged with an argon for 20 min. to remove the dissolved oxygen and the argon blanket was kept to prevent oxygen diffusion into the solution. The measurements were done in three-electrode configuration using the platinum electrode or modified ITO electrode as a working electrode, the non-aqueous Ag/Ag^+^ electrode as a reference electrode and platinum wire as a the auxiliary electrode. The ferrocene was used as an external standard and the cyclic voltammograms were calibrated against Fc/Fc^+^ redox couple. Spectroelectrochemical measurements were done using a potentiostat connected to a Jasco V-770 UV-Vis-NIR spectrometer (Tokyo, Japan) using commercially available honeycomb electrode and the non-aqueous Ag/Ag^+^ electrode as a reference electrode calibrated against ferrocene. Thermogravimetric analysis (TGA) was carried out using a TGA 4000, PerkinElmer analyzer (Shelton, CT, USA) in the temperature range of 30 °C–650 °C at a heating rate of 10 °C/min in nitrogen.

Electrochromice device was constructed according to the previously described method [4].

### 3.1. Synthesis of Monomer

Under an argon atmosphere, 1,4,5,8-naphthalenetetracarboxylic dianhydride (0.200 g, 0.75 mmol) was solubilized in an anhydrous dimethylformamide (DMF, 2 mL) and 4-vinylaniline (0.165 g, 1.65 mmol) was added to the solution. The reaction was conducted in a pressure-resistant, tightly closed reaction vessel. The process was conducted in a Discovery 2.0 CEM microwave reactor (CEM Corporation, Matthews, NC, USA), maintaining a temperature of 160 °C for 1.5 h. After completing the reaction, the reaction mixture was poured into a 1.0 M hydrochloric acid solution, which caused the product to precipitate. The precipitate was filtered off, washed with distilled water, and dried. A total of 215 mg of a dark solid was obtained (yield: 78%). ^1^H NMR (600 MHz, DMSO) δ 8.71 (s, 4H), 7.66 (d, *J* = 8.4 Hz, 4H), 7.44 (d, *J* = 8.3 Hz, 4H), 6.85 (dd, *J* = 17.7, 11.0 Hz, 2H), 5.95 (dd, *J* = 17.7, 0.9 Hz, 2H), 5.38 (dd, *J* = 10.9, 0.9 Hz, 2H) ppm. ^13^C NMR (150 MHz, DMSO) δ 163.4, 137.8, 136.5, 135.5, 130.9, 129.7, 127.5, 127.1, 115.9 ppm. EI-MS *m*/*z* = 470 (M^ꞏ+^). Elemental analysis calcd. for C_30_H_18_N_2_O_4_ (470.1267) calcd. C, 76.59; H, 3.86; N, 5.95; found C, 76.57; H, 3.89; N, 5.94 %.

### 3.2. Photopolymerization

ITO glass electrode was cleaned by sonication in water and 2-propanol for 15 min. Afterwards, it was exposed to an UV-ozone atmosphere for 20 min. The monomer (4 mg) and 2,2-dimethoxy-2-phenylacetophenone (~10% mol) were dissolved in dichloromethane:methanol solvent mixture 5:1 (*v*/*v*) and the solution was spray-coated on the substrate. The substrate was irradiated with a UV light at 365 nm for 10 min using a PhotoCube photoreactor (ThalesNano, Budapest, Hungary). The polymerized layers were rinsed with dichloromethane to remove unreacted monomers and low molecular weight oligomers, and dried on air.

## 4. Conclusions

In summary, we have successfully synthesized the NDI–styrene monomer via a simple microwave-assisted condensation reaction. The monomer was polymerized onto the substrate using a photopolymerization protocol, which allowed for the formation of a pattern on the substrate. FT-IR spectroscopy showed that the peak corresponding to the stretching vibrations of the carbon–carbon double bond of the vinyl group from the styrene moiety disappeared, confirming the successful polymerization of the monomer on the substrate. Both the monomer and polymer were characterized by cyclic voltammetry, and their electrochromic properties were investigated. The prototype electrochromic device has been fabricated using the NDI-based polymer as an active material, and its performance has been examined. During electrochromic processes, the film exhibited a color change from yellow to brown-red to green. This was the result of the formation of a radical anion and dianion in the NDI core. The electrochromic device could be operated continuously for 5.5 h. The research conducted here opens up the possibility of the formation of electrochromic polymers of good electrochromic performance via the photopolymerization protocol.

## Figures and Tables

**Table 1:** Comparison of electrochromic properties of the investigated polymer and other NDI-based materials.

Compound	Switching Times		ΔT%	Coloration Efficiency [cm^2^/C]	Reference
	t_c_	t_b_			
NDI-based polymer	18 s	6 s	26%	238	This work
Zn-NDI MOF	3 s	91 s	21%	117	[85]
Disodium salt of *N*,*N*′-bis(4-benzosulfonic acid)NDI	16.5 s	15.8 s	56%	260	[44]
Thiophene–NDI donor-acceptor polymer	-	-	23%	82	[86]
Zr-NDI MOF	49 s	35 s	39.7%	158	[47]

## Data Availability

The original contributions presented in this study are included in the article and Supplementary Materials.

## Supplementary Materials

The following supporting information can be downloaded at: https://www.mdpi.com/article/10.3390/ijms26104807/s1.
