# Supplementary material for: Photopolymerization of Styrene–Naphthalenediimide Monomer: Formation of Pattern and Electrochromism
Source: Int J Mol Sci. 2025 May 17;26(10):4807. doi: 10.3390/ijms26104807 (PMC12111903; doi:10.3390/ijms26104807)
Supplement: Supplementary file 1 [file ijms-26-04807-s001.zip › ijms-3589268-supplementary.pdf]

Supplementary information

Photopolymerization of styrene-naphthalenediimide monomer: formation of pattern and electrochromism

Marcin Nowacki, Marcin Hoffmann, Monika Wałęsa-Chorab*
Faculty of Chemistry, Adam Mickiewicz University in Poznań, Uniwersytetu Poznańskiego 8, 61-614 Poznań, Poland
* *Correspondence: mchorab@amu.edu.pl*

Table of content

Figure S 1. The preparation of the NDI-based monomer. 3
Figure S 2. ^{1}H NMR spectra of NDI-styrene monomer in d_6-DMSO. 3
Figure S 3. ^{13}C NMR spectra of NDI-styrene monomer in d_6-DMSO. 4
Figure S 4. EI-MS spectra of NDI-styrene monomer. 4
Figure S 5. TGA curves for styrene-NDI monomer and polymer. 5
Figure S 6. Optimized geometry of NDI-styrene monomer and its simulated (TD-DTF) UV-Vis spectrum. 5
Figure S 7. Obtaining of the absorption edge wavelength of the NDI-styrene monomer. 6
Figure S 8. Obtaining of the absorption edge wavelength of the NDI-based polymer. 6
Figure S 9. Cyclic voltammograms of polymer NDI obtained at different scan rates 7
Figure S 10. Linear dependence of cathodic current on the scan rate. 7
Figure S 11. UV-Vis spectroelectrochemistry of NDI-styrene polymer immobilized on ITO electrode measured in 0.1 M solution of $TBAClO_4$ in acetonitrile. 8
Figure S 12. The optical memory of the device fabricated using poly-NDI as an active layer. 8

Table S 1. Solubility test of the monomer in different organic solvents. 8
Table S 2. Cartesian coordinates of the optimized geometry 9

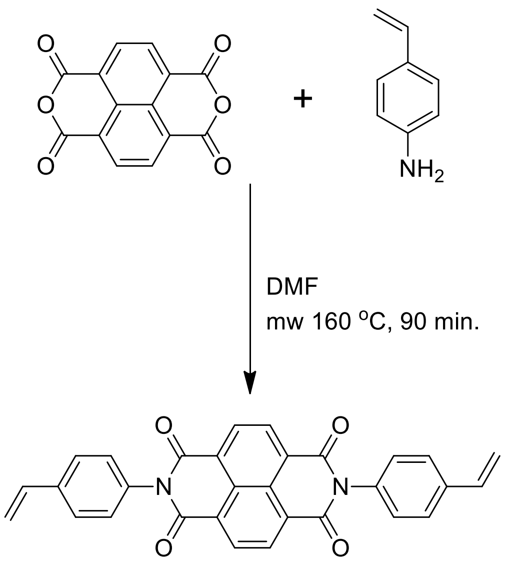

Figure S 1. The preparation of the NDI-based monomer.

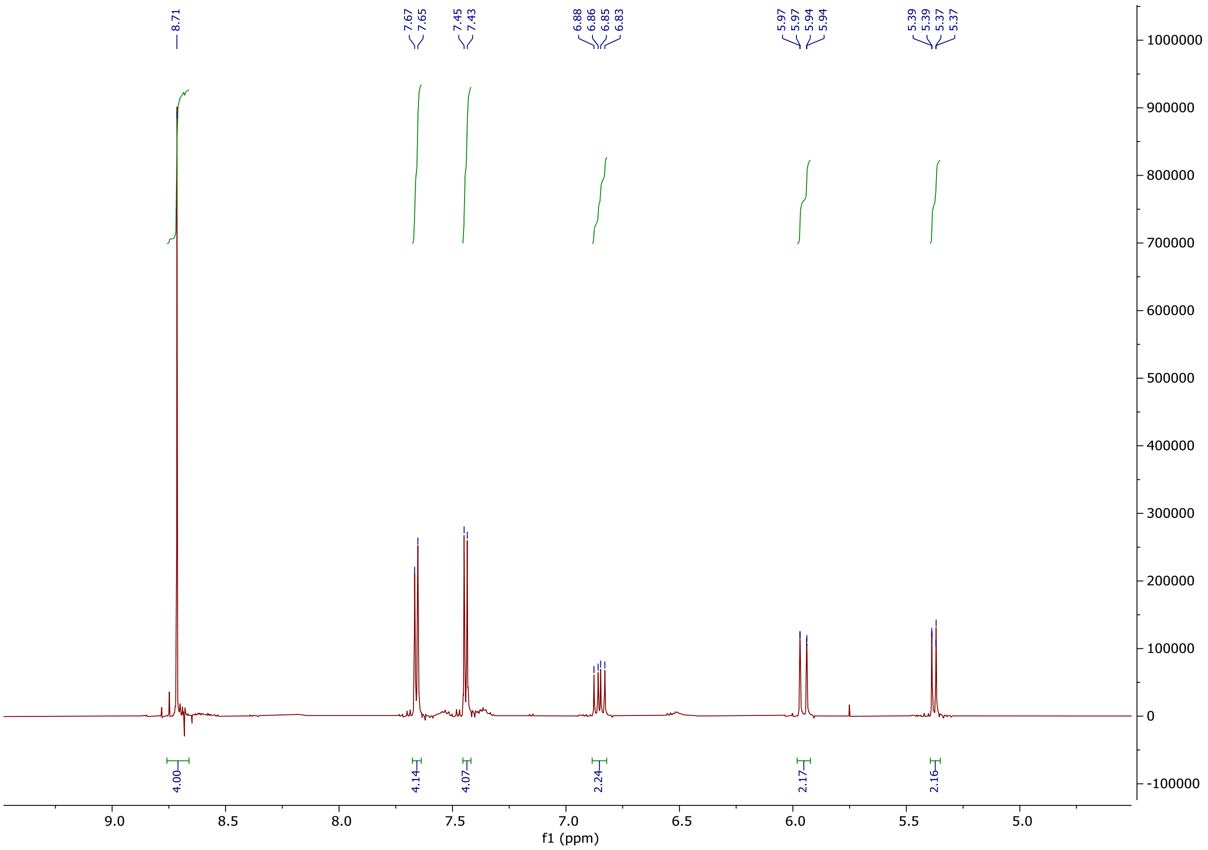

Figure S 2. ^{1}H NMR spectra of NDI-styrene monomer in d_6-DMSO.

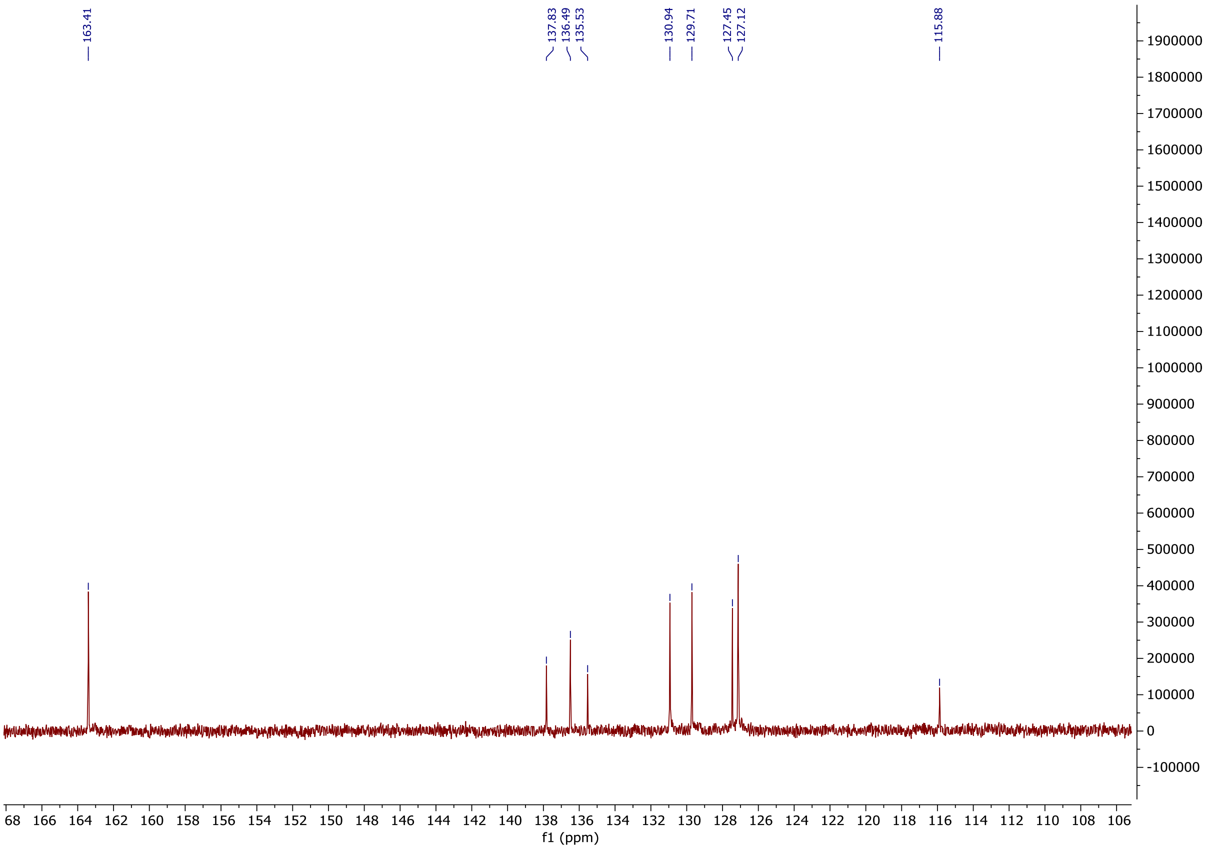

Figure S 3. ^{13}C NMR spectra of NDI-styrene monomer in d_6-DMSO.

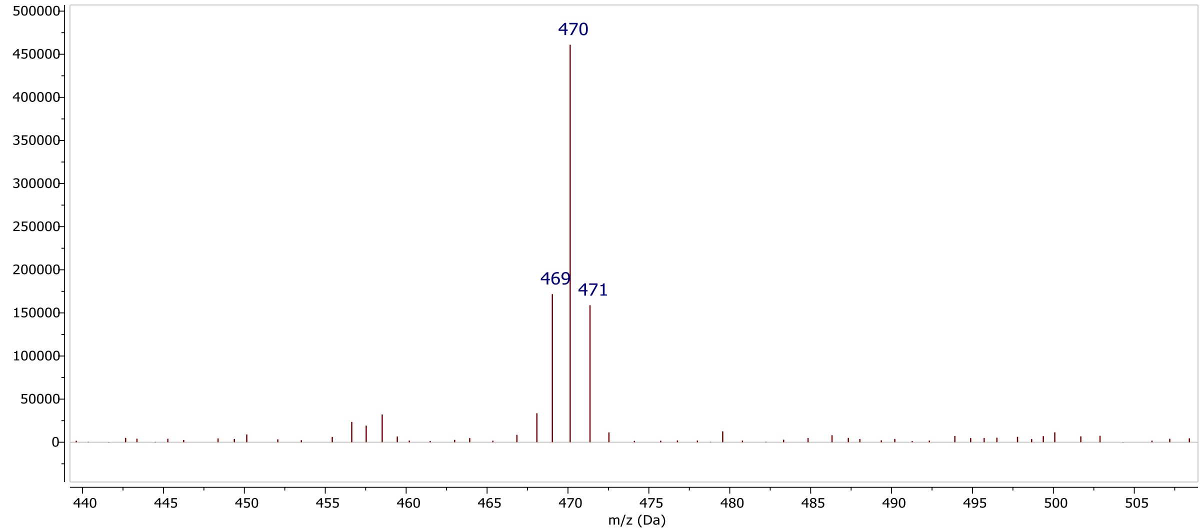

Figure S 4. EI-MS spectra of NDI-styrene monomer.

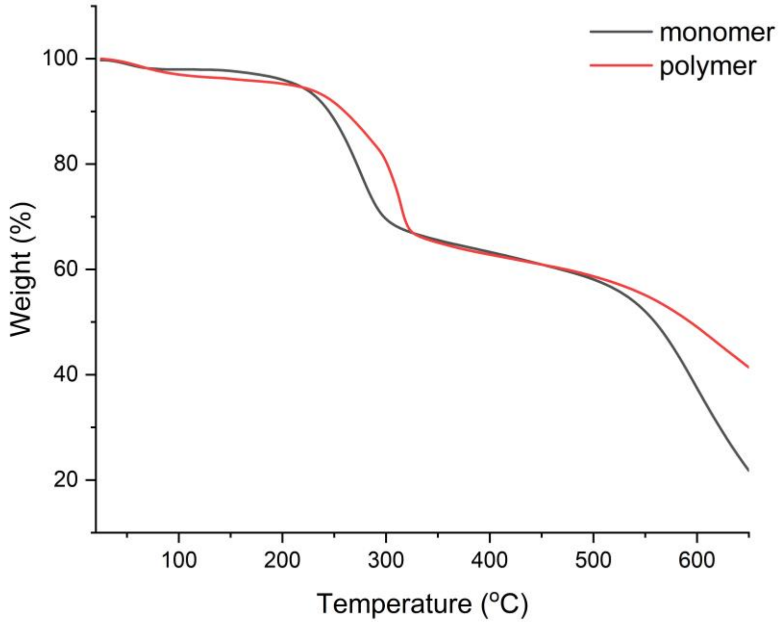

Figure S 5. TGA curves for styrene-NDI monomer and polymer.

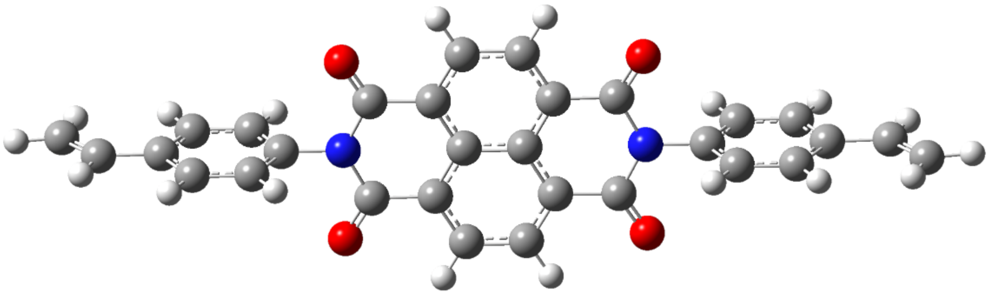

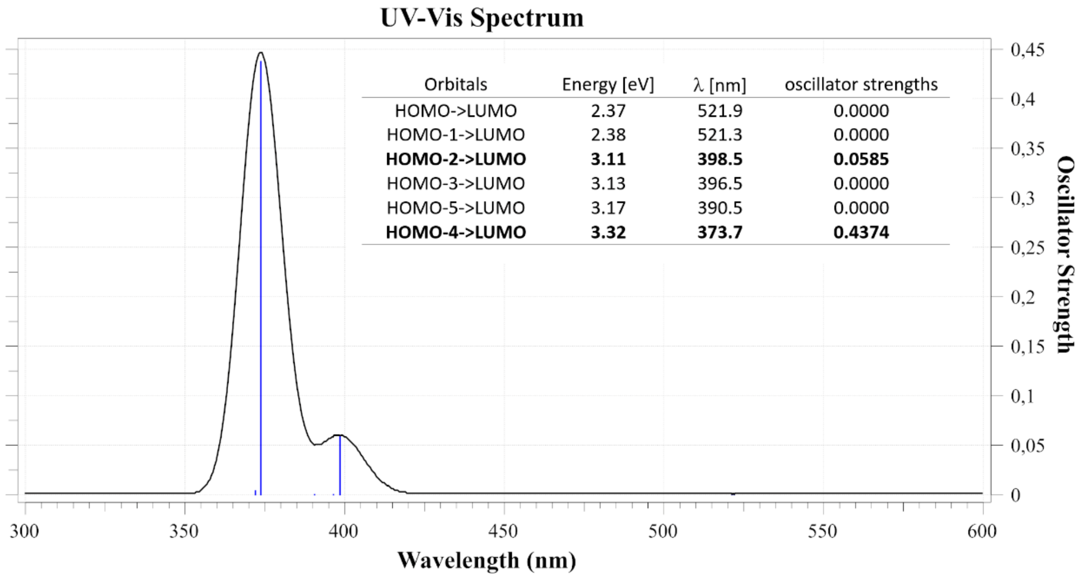

Orbitals	Energy [eV]	λ [nm]	oscillator strengths
HOMO->LUMO	2.37	521.9	0.0000
HOMO-1->LUMO	2.38	521.3	0.0000
**HOMO-2->LUMO**	**3.11**	**398.5**	**0.0585**
HOMO-3->LUMO	3.13	396.5	0.0000
HOMO-5->LUMO	3.17	390.5	0.0000
**HOMO-4->LUMO**	**3.32**	**373.7**	**0.4374**

Figure S 6. Optimized geometry of NDI-styrene monomer and its simulated (TD-DTF) UV-Vis spectrum.

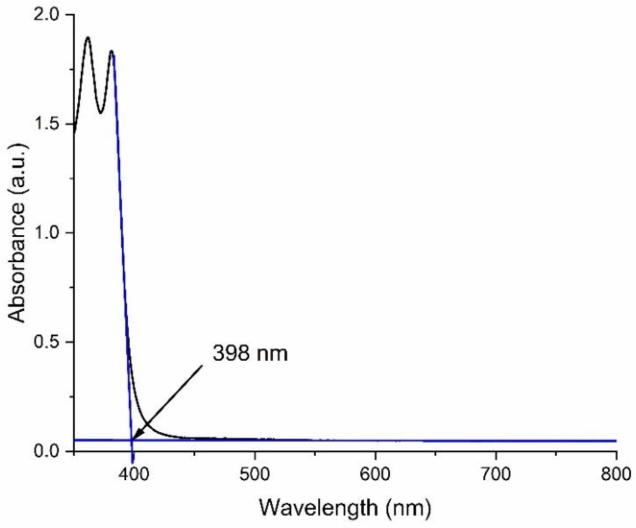

Figure S 7. Obtaining of the absorption edge wavelength of the NDI-styrene monomer.

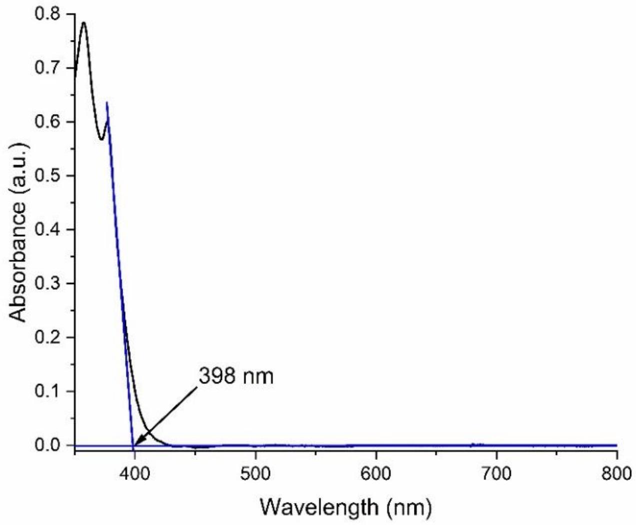

Figure S 8. Obtaining of the absorption edge wavelength of the NDI-based polymer.

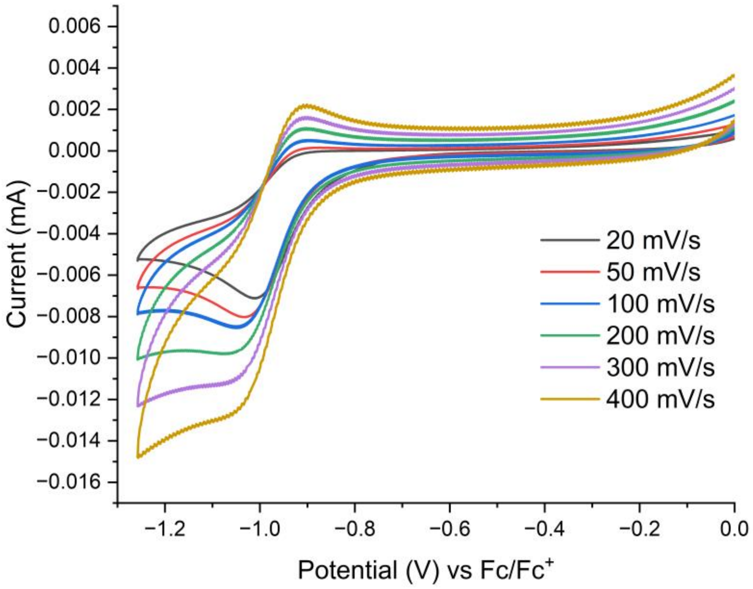

Figure S 9. Cyclic voltammograms of polymer NDI obtained at different scan rates.

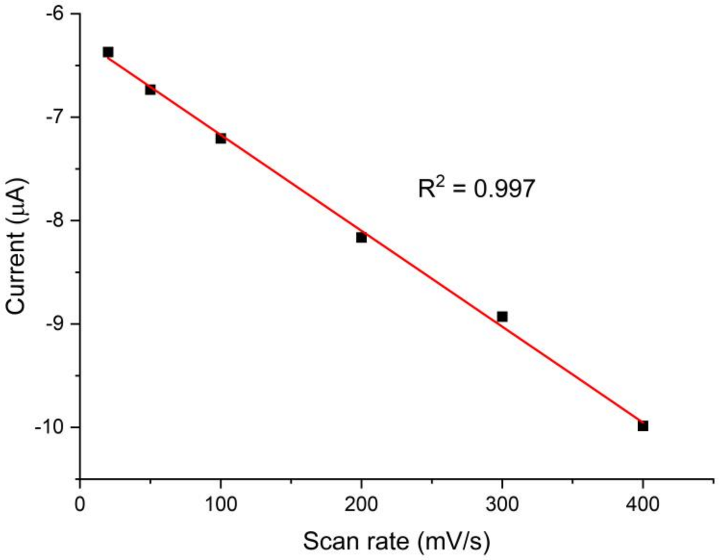

Figure S 10. Linear dependence of cathodic current on the scan rate.

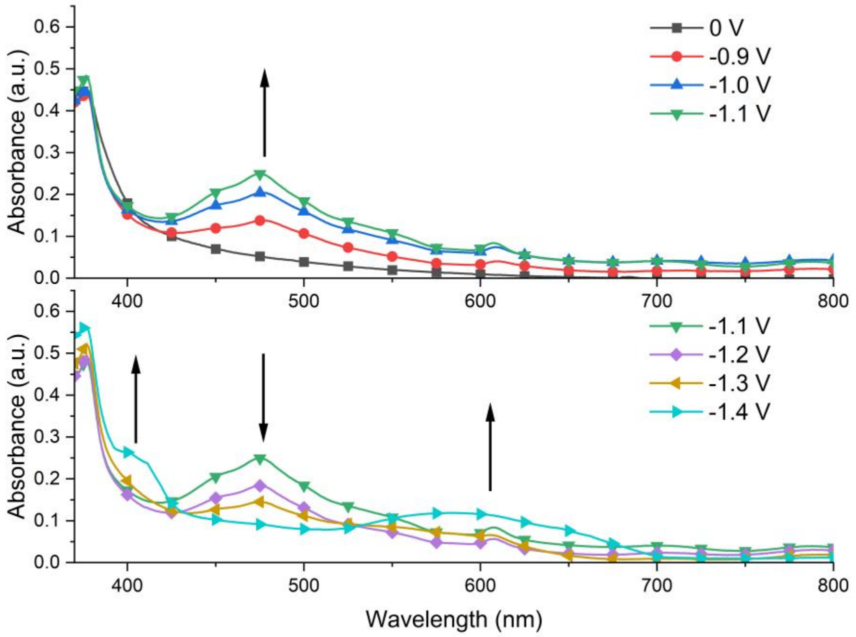

Figure S 11. UV-Vis spectroelectrochemistry of NDI-styrene polymer immobilized on ITO electrode measured in 0.1 M solution of $TBAClO_4$ in acetonitrile.

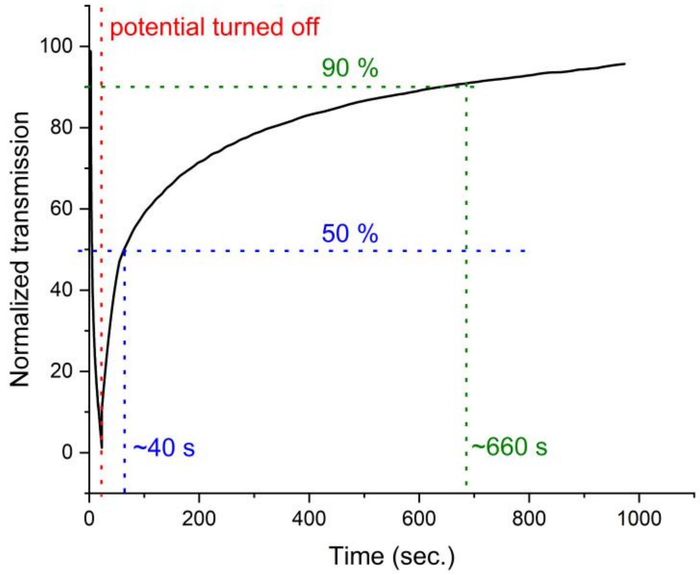

Figure S 12. The optical memory of the device fabricated using poly-NDI as an active layer.

Table S 1. Solubility test of the monomer in different organic solvents.

Solvent	Solubility of NDI monomer
Ethyl acetate	insoluble
Tetrahydrofuran	insoluble
Dichloromethane	insoluble
Methanol	insoluble

Acetonitrile	insoluble
Dimethylsulfoxide (DMSO)	insoluble
Dimethylformamide (DMF)	insoluble
Dichloromethane:mathanol 5:1 (v/v)	insoluble
Dichloromethane methanol 1:1 (v/v)	insoluble

Table S 2. Cartesian coordinates of the optimized geometry

C	1.406866	1.234755	-0.030723
C	0.711623	0.000007	-0.015598
C	1.406887	-1.234729	-0.030680
C	0.704062	2.430133	-0.015371
C	-0.711623	-0.000004	0.015600
C	-1.406887	1.234732	0.030671
C	-0.704105	2.430121	0.015246
C	-1.406865	-1.234752	0.030731
C	-0.704062	-2.430130	0.015369
C	0.704105	-2.430119	-0.015257
H	1.259475	-3.361694	-0.027303
H	-1.259415	-3.361715	0.027446
H	1.259416	3.361718	-0.027444
H	-1.259475	3.361697	0.027281
C	-2.894986	-1.253891	0.063379
O	-3.536437	-2.292018	0.076378
C	-2.895007	1.253847	0.063345
O	-3.536469	2.291969	0.076432
C	2.895006	-1.253845	-0.063385
O	3.536468	-2.291965	-0.076474
C	2.894987	1.253894	-0.063341
O	3.536436	2.292021	-0.076429
N	3.542474	0.000028	-0.079091
N	-3.542474	-0.000025	0.079113
C	-4.991551	-0.000035	0.111533
C	-5.657798	0.000015	1.333599
C	-5.711406	-0.000067	-1.084470
C	-7.051580	0.000037	1.354861
H	-5.091803	0.000061	2.259692
C	-7.101835	-0.000033	-1.055620
H	-5.181586	-0.000138	-2.031859
C	-7.801841	0.000021	0.165871
H	-7.569133	0.000083	2.310314
H	-7.645769	-0.000045	-1.994440
C	4.991551	0.000037	-0.111518
C	5.711407	0.000032	1.084484

C	5.657797	0.000026	-1.333585
C	7.101837	-0.000003	1.055633
H	5.181588	0.000075	2.031873
C	7.051578	0.000003	-1.354847
H	5.091801	0.000010	-2.259678
C	7.801841	-0.000020	-0.165858
H	7.645771	-0.000021	1.994453
H	7.569131	-0.000014	-2.310301
C	-9.272624	0.000079	0.253312
C	-10.151224	-0.000046	-0.759900
H	-9.664723	0.000250	1.269718
H	-11.218939	0.000029	-0.566891
H	-9.849386	-0.000217	-1.803226
C	9.272624	-0.000077	-0.253302
C	10.151225	0.000010	0.759909
H	9.664721	-0.000213	-1.269709
H	11.218940	-0.000059	0.566899
H	9.849388	0.000145	1.803235
